# Control of *Mycobacterium avium subsp. paratuberculosis* infection on a New Zealand pastoral dairy farm

**DOI:** 10.1186/s12917-019-2014-6

**Published:** 2019-07-29

**Authors:** Andrew Bates, Rory O’Brien, Simon Liggett, Frank Griffin

**Affiliations:** 1 Vetlife Centre for Dairy Excellence, Vetlife Scientific, 1 Waitohi-Temuka Road, Temuka, 20 Wilson Street, Geraldine, New Zealand; 2 Disease Research Limited, Invermay Agricultural Centre, Mosgiel, New Zealand; 3 Department of Microbiology & Immunology, University of Otago, Dunedin, New Zealand

**Keywords:** Johne’s disease, Control program, ELISA, Quantitative PCR

## Abstract

**Background:**

Johne’s disease is a major production limiting disease of dairy cows caused by infection with *Mycobacterium avium subsp. paratuberculosis* in calf-hood. The disease is chronic, progressive, contagious and widespread with no treatment and no cure. Economic losses arise from decreased productivity through reduced growth, milk yield, fertility and also capital losses due to premature culling or death. Control chiefly centers upon removing those animals which actively shed bacteria and protecting calves from infection. A prolonged pre-clinical shedding phase, lack of test sensitivity, organism persistence and abundance in the environment as well as management systems that expose susceptible calves to infection make control challenging, particularly in pastoral, seasonal dairy systems. Combining a novel testing strategy to remove infected cows along with limited measures to protect vulnerable calves at pasture, this study reports the successful reduction over a four-year period of seroprevalence of cows testing positive for MAP infection in a New Zealand pastoral dairy herd.

**Results:**

For all age groups considered the apparent seroprevalence of cows testing positive decreased from 297 / 1,122 (26%) in 2013–2014, to 24 / 1,030 (2.3%) in 2016–2017. Over the same period, the apparent seroprevalence in primiparous cows decreased from 39 / 260 (15%) to 7 / 275 (2.5%) and in multiparous cows from 258 / 862 (29.9%) to 17 / 755 (2.3%). The reported proportion of calved cows culled annually from suspected clinical Johne’s disease fell from 55 / 1,201 (5%) in the year preceding the control program to 5 / 1,283 (0.4%) in the final year of the study.

**Conclusions:**

On this farm**,** reduction in the prevalence of infection was achieved by reducing the infectious pressure through targeted culling of heavily shedding animals together with limited measures to protect calves at pasture from exposure to *Mycobacterium avium subsp. paratuberculosis*. Whilst greater protection of young animals through separation from infected cows and their colostrum and milk would have reduced the risk of neonatal infection, this study demonstrates, in this case, that these management measures while prudent were not essential for effective reduction in the prevalence of MAP infection.

## Background

Johne’s disease (JD) is a chronic disease of ruminant species caused by intestinal infection with *Mycobacterium avium subsp. paratuberculosis* (MAP). Infection with MAP is predominantly subclinical in most dairy cows with farmers becoming aware of the disease when the clinical signs of infection such as diarrhea and wasting become apparent [1]. Whitlock and Buergelt [2] suggested a bovine JD “iceberg effect” whereby, for every clinically affected animal born on the farm, a minimum of 25 other animals are likely to be infected.

A prolonged incubation period of typically 4–5 clinically normal years following calf-hood infection typically precedes production and weight loss, diarrhea and death [3]. During the clinically normal period, infected cows shed MAP in their feces and their milk thus transmitting the organism to multiple generations within the herd and contaminating the environment [1] where the organism can persist for many months [4]. Shedding increases and can include transplacental spread as clinical signs develop [5]. This has led to classification of 3 disease states - Infected (but not yet shedding or showing clinical signs), Infectious (infected and shedding but not yet showing clinical signs) and Affected (infected, shedding and showing clinical signs) [6, 7].

Within New Zealand (NZ), Norton et al. (2009) reported that 47% of 427 North Island dairy farmers surveyed had suspected clinical JD in their herds within the previous 5 years. In a NZ-wide survey of 551 dairy farmers, Hunnam (2014) found an average herd prevalence of 54.3% (43.5% for the North Island and 67.8% South Island) based on farmer diagnosis. Average incidence within these herds was 0.47% (range 0–6.2%). International studies based on serological or fecal surveys indicate a similar picture of low levels of clinical disease (< 5%) but with higher overall herd prevalence (50–70%) [3, 4, 8]. Differences in diagnostic sensitivity and specificity confound comparison between studies, particularly when comparing farmer reporting of clinical disease with laboratory screening of herds [7].

Control of JD on dairy farms rests on a layered approach centred upon reducing the spread of infection within the herd (biocontainment) [1]. This involves identifying animals that are infectious and reducing the spread of infection to calves, coupled with decreasing the risk of importing infected animals (bioexclusion) [9]. This approach has been well validated overseas [1] but farmers have been slow to adopt these methods under NZ seasonal and pastoral farming systems [10, 11]. In part this arises because farmers’ awareness of the impact of the disease is typically confined to the end stage clinical signs (diarrhea and weight loss) rather than the pre-clinical effects on herd production [11]. Effective identification of infectious animals prior to this stage is a vital part of on farm control; this is often considered difficult to achieve, however, because of the low specificity and sensitivity of many currently available diagnostic tests [7], particularly for the pre-clinical forms of the disease [3].

The specificity of ELISA tests may be compromised by common antigens shared between MAP, *Mycobacterium avium* and other saprophytic environmental mycobacteria. The sensitivity of ELISA tests, particularly for sub-clinically infected animals in the early stages of JD, is also influenced by the dynamics of antibody production [12] and the stage of disease [6]. In their recent evaluation of MAP testing strategies, More et al. (2015) estimated that a single serum ELISA for MAP had a sensitivity of 0.15 in Infected animals, 0.47 in Infectious animals and 0.71 in Affected animals. While detection of the organism via fecal culture on Herrold’s egg yolk medium has been a definitive test for MAP infection this requires prolonged incubation periods of up to 16 weeks and may be compromised by overgrowth by contaminating gut organisms [13–15]. Internationally, the rapid, direct and quantitative measurement of MAP shedding in feces of infected and affected animals by quantitative PCR is rapidly becoming the standard and widely used method for JD diagnostic testing [16–20].

Management decisions to protect calves from infection such as separation of infected cows at calving and discard of calves, milk and colostrum from MAP positive cows, or pasteurisation of their milk, are uncommon in seasonal, pastoral NZ dairy farming. Pasture management is greatly complicated by any increase in the number of groups of grazing cows [21]. The NZ Animal Compounds and Veterinary Medicines act (1987) prohibits the sale of milk for human consumption when that milk is contaminated with drug residues. Consequently, calves are commonly fed on milk from sick cows, those undergoing antimicrobial treatment or excluded from the main herd for other reasons.

Therefore, in NZ, there has been relatively little engagement from dairy farmers in the control of JD unless they have experienced a high clinical prevalence [8] and there is evidence of increasing prevalence of JD [8, 9] especially in the South Island of the country [9].

In this situation, we report the results of a single herd study where a high prevalence of clinical JD and MAP infection has been reduced over a 4 year period using an annual test and cull approach [16, 22]. This strategy is based on a herd testing protocol using an initial herd screening using serological ELISA for multiple MAP antigens [22] coupled with a quantitative fecal PCR (fPCR) test to confirm the status of ELISA positive animals [23, 24]. This approach allows farmers and their advisers to stratify shedders according to disease status and environmental risk.

Our null hypothesis was that the prioritized removal of the highest shedders would facilitate the early removal of animals contributing to the greatest level of environmental contamination with MAP bacteria. This would reduce infection pressure and allow alternative risk mitigation options to be implemented for low shedders. The broad dynamic range of fPCR detection of MAP also lent itself well to pooled sampling as high shedding individuals may be easily identified amongst low or non-shedders even at considerable dilution [25]. In this way, pooled screening of samples with fPCR greatly reduced the cost compared to whole herd fPCR testing.

## Results

Over the 4 year period a total of 4,358 blood samples were submitted from 2,211 cows and of these 683 were submitted for fPCR. The change in seroprevalence for JD over the 4 seasons and a summary of the independent variables is given in Table 1.

**Table 1:** Median, 10th and 90th centile of milk solids production, age and days in milk for cows from a NZ, pastoral dairy farm which underwent annual screening for infection with MAP. Infection status was assessed using whole herd serum ELISA. ELISA results were classified as Not Detected (< 50 ELISA units (EU)), Low (50–100 EU), Moderate (101–150 EU) or High (> 150 EU). Results are presented as numerical count and proportions together with 95% confidence intervals. Results with differing superscripts are statistically different (*p* <  0.05)

Season	2013–2014			2014–2015			2015–2016			2016–2017		
Variable (centile)	10th	50th	90th	10th	50th	90th	10th	50th	90th	10th	50th	90th
Age (years)	3	5	8	3	5	8	3	5	8	3	5	8
Days in Milk	215	258	268	219	255	267	221	265	269	218	259	268
Proportion Friesian	0.50	0.75	1.00	0.50	0.75	1.00	0.50	0.75	1.00	0.5	0.75	1.0
Cows ELISA tested	1,122			1,069			1,137			1,030		
	Number (Proportion)	95% CI		Number (Proportion)	95% CI		Number (Proportion)	95% CI		Number (Proportion)	95% CI	
ELISA Not Detected	825 (0.74)^a^	0.71–0.76		961 (0.90)^b^	0.88–0.95		1,062 (0.93)^c^	0.92–0.95		1,006 (0.98)^d^	0.97–0.99	
ELISA Low	157 (0.14)^a^	0.12–0.16		39 (0.04)^b^	0.03–0.05		33 (0.03)^b^	0.02–0.04		6 (0.01)^c^	0.00–0.01	
ELISA Moderate	63 (0.06)^a^	0.04–0.07		27 (0.03)^b^	0.02–0.03		19 (0.02)^b^	0.01–0.03		2 (0.00)^c^	0.00–0.00	
ELISA High	77 (0.07)^a^	0.05–0.08		42 (0.04)^b^	0.03–0.05		23 (0.02)^c^	0.01–0.03		16 (0.02)^c^	0.01–0.02	

### Culling

**Fig. 1:**
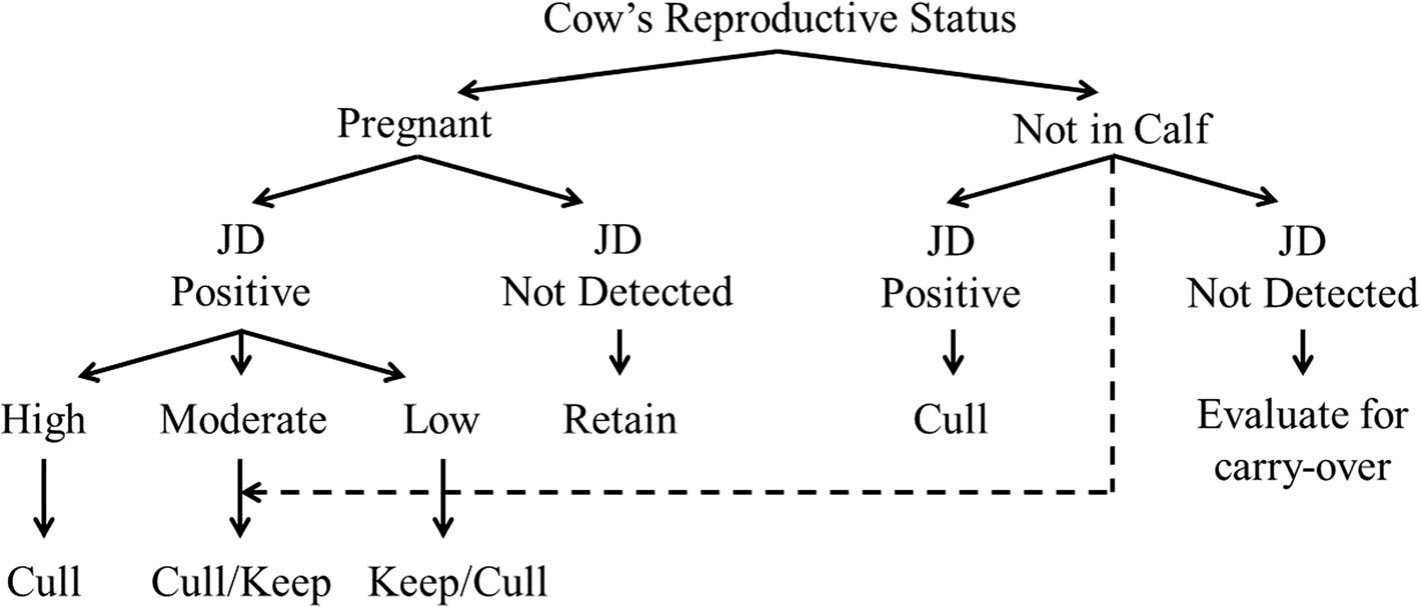
Decision tree used to determine retention-cull at the end of each season

Culling was defined as an unplanned exit from the herd during the study period. It included cows sold off farm for slaughter for human consumption, cows slaughtered for salvage value and cows that died on farm [26]. The reasons for culling are often poorly recorded on commercial farms and estimates from single farm studies can be unreliable [27]. However, over the study period the proportion of calved cows culled annually with suspected clinical JD (based on veterinary clinical diagnosis of a non-pyrexic cow, with diarrhea for more than 5 days, losing body condition despite a normal appetite) fell from 5% (55 / 1,201) in the year preceding the control program to 0.4% (5 / 1,283) in the final year of the study, (*p* <  0.001). For each season, culling decisions followed a decision tree approach outlined in Fig. 1. To aid the removal of animals shedding large numbers of MAP, a priority was made to remove all animals with a high fPCR status, followed by those that were ELISA High [28]. In Fig. 1, a dotted line indicates that retention of pregnant cows testing Medium or Low for JD was determined on an individual cow basis driven by the herd’s not in calf rate and individual cow factors (age, clinical mastitis history and somatic cell count, lameness record, production and temperament).

At the end of the 2013–2014 season, 77 / 77 ELISA High, 46 / 63 ELISA Moderate and 0 / 157 ELISA Low cows were culled, such that of 386 animals culled, 123 were JD positive (78 ELISA positive only and 45 both ELISA and fPCR positive). At the end of the 2014–2015 season, 42 / 42 ELISA High, 2 / 27 ELISA Moderate and 0 / 39 ELISA Low cows were culled such that of 375 cows culled, 44 were JD positive (27 ELISA positive only and 17 both ELISA and fPCR positive). At the end of the 2015–2016 season, 21 / 23 ELISA High, 0 / 19 ELISA Moderate and 0 / 33 ELISA Low cows were culled amongst the 371 cows removed (such that of the 21 JD positive cows, 11 were ELISA positive only and 10 both ELISA and fPCR positive). At the end of the 2016–2017 season, because the proportion of ELISA positive cows overall was low, all were culled (16 / 16 High, 2 / 2 Moderate and 6 / 6 Low) such that of the 390 cows culled, 24 were JD positive (ELISA only as fPCR was not performed in that year as all ELISA positive cows were culled).

The herd remained closed for the duration of the study and management changes 2, 4, 5, 6, and 7 listed in Table 2 were implemented on farm. Management changes 1 and 3 listed in Table 2 (requiring that replacement heifers, colostrum and calf milk were taken only from animals with a negative JD status) were not implemented by the farmer. This meant that replacement heifers, colostrum and calf milk were sourced from all animals regardless of JD status.

**Table 2:** Management changes suggested to reduce the prevalence of Johne’s disease in a New Zealand, pastoral dairy herd infected with MAP

Management change required	
1. Separation of all ELISA positive cows 1 month before and during calving.	
2. No calves to be retained as heifer replacements if born to ELISA positive dams.	
3. Colostrum and milk from all ELISA positive dams not to be fed to any replacement calves.	
4. Calves to be housed in calf pens and physically separated from cows within 24 h of birth.	
5. Calves to be grazed on paddocks to which adult cows (≥ 2 years) have not had access.	
6. All cows to be annually blood tested in the autumn using a modified ELISA test with fPCR used to confirm the status of ELISA positive animals.	
7. All animals testing high for the ELISA or fPCR to be culled from the herd before the end of the current lactation.	
8. As many as possible additional but lower grade ELISA and fPCR positive cows to be included on the herd’s annual cull list.	

### ELISA status

For all age groups the apparent prevalence of cows testing positive (≥ 50 EU in any one of 4 ELISA tests conducted in parallel) decreased from 297 / 1,122 (26%) in 2013–2014 to 24 / 1,030 (2.3%) in 2016–2017 (*p* < 0.001). Over the same period, the apparent prevalence decreased from 39 / 260 (15%) in primiparous cows to 7 / 275 (2.5%; *p* < 0.001) and in multiparous cows from 258 / 862 (29.9%) to 17 / 755 (2.3%; *p* < 0.001). The change in apparent prevalence from 2014 to 2017 of ELISA positive, primiparous and multiparous animals is detailed in Table 3.

**Table 3:** Change in apparent sero-prevalence from 2014 to 2017 of ELISA positive, primiparous and multiparous animals in a study of MAP infection in a South Canterbury dairy herd over 4 years of intervention

Season	Primiparous cows in herd	Multiparous cows in herd	Animals seropositive for JD	
			Percentage (95% CI) Primiparous animals	Percentage (95% CI) Muliparous animals
2013–2014	260	862	15 (10.7–19.3)	30 (26.9–33.0)
2014–2015	336	733	4 (1.8–5.9)	13 (10.5–15.4)
2015–2016	327	810	7 (4.5–10.2)	6.3 (4.6–8.0)
2016–2017	275	755	2.6 (1.0–4.4)	2.3 (1.2–3.3)

**Fig. 2:**
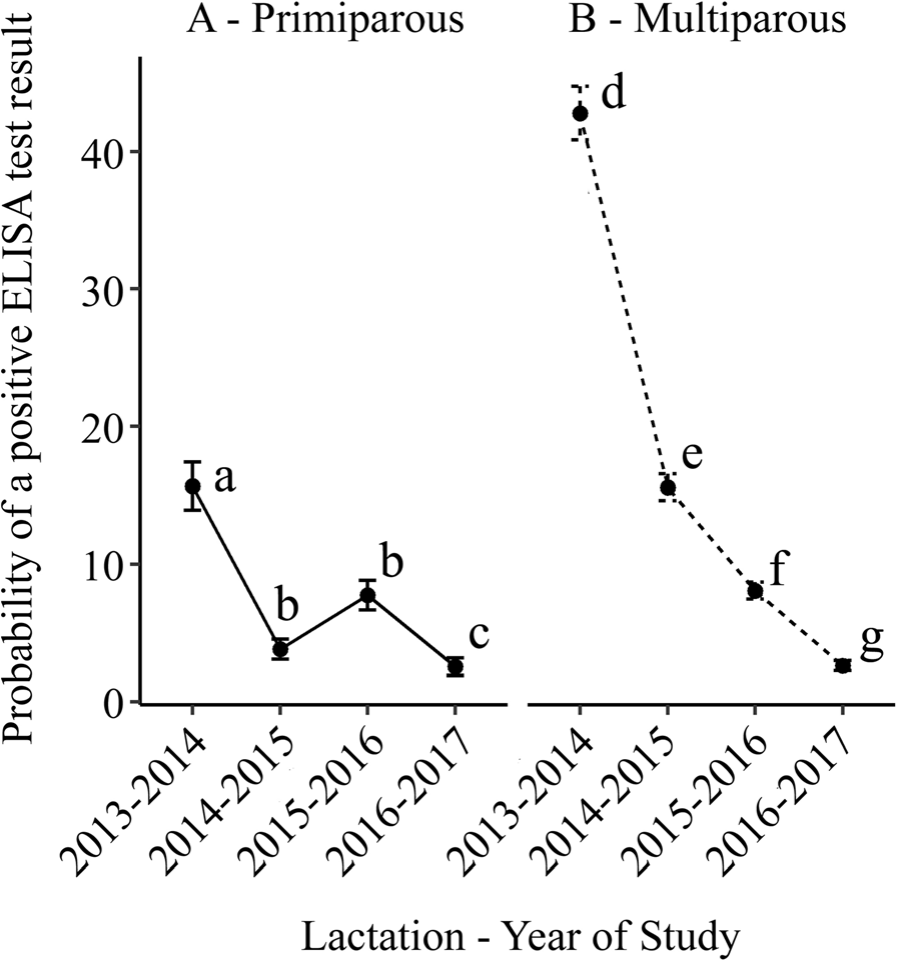
Predicted probability of a positive ELISA test result (positive > = 50 EU in any one of 4 ELISA tests conducted in parallel) for primiparous (left hand panel) and multiparous cows (right hand panel) in an analysis of the association between ELISA status for *Mycobacterium avium subsp.* paratuberculosis infection and age and year of study on a New Zealand pastoral dairy farm over four seasons (2013-2017)

For the first 2 seasons the GEE predicted that multiparous cows were more likely to be ELISA positive than primiparous cows. The odds for being ELISA positive decreased with each year of the study for multiparous cows but there was an interaction between age and study year (*p* < 0.001) when age was dichotomized into primiparous and multiparous. The effect of the interaction with year was for heifers to be less likely to be ELISA positive in each subsequent year of the study except for 2015–2016. The results for the GEE model for ELISA status are presented in Table 4 and the predicted probability of a positive ELISA for primiparous and multiparous cows in Table 5 and in Fig. 2. In Fig. 2, within each parity group, different letters indicate statistically significant differences (*p* < 0.05) between study years. Across all 4 years of the study, there was a significant interaction between parity and year (*p* < 0.001) such that for each year of the study, primiparous and multiparous cows were less likely to be ELISA positive except for 2015–2016, where the probability of positive ELISA status increased for primiparous cows.

**Table 4:** Results for the general estimating equation predicting positive ELISA status (≥ 50 EU) in any one of 4 ELISA tests conducted in parallel) for an analysis of the association between ELISA status for MAP infection and age and year of study on a NZ dairy farm over 4 seasons (2013–2017)

Input variable	Coefficient	Odds Ratio (OR)	95% CI	*p*-value^a^
Parity^b^				
Primparous	Ref	Ref		
Multiparous	0.62	1.86	1.41–2.45	< 0.001
Study year				
2013–2014	Ref	Ref		
2014–2015	−1.41	0.24	0.12–0.49	< 0.001
2015–2016	0.71	2.03	0.98–4.19	0.055
2016–2017	−1.11	0.33	0.14–0.77	0.010
Interaction ^d^				
Multiparous 2014–2015	0.41	1.50	0.72–3.12	0.284
Multiparous 2015–2016	−1.35	0.26	0.12–0.56	< 0.001
Multiparous 2016–2017	−0.02	0.98	0.41–2.34	0.970
ICC^e^		0.38	0.35–0.42	
Variance within		0.063		
Variance among		0.039		

^a^Significance of coefficient

^b^Parity of cow defined as primiparous (≤ 2 years) and multiparous (> 2 years)

^c^Milking season. Each season uses the preceding season as the referent

^d^Interaction term between parity and milking season. Overall significance of the interaction term: < 0.001

^e^Intra class correlation coefficient

**Table 5:** GEE prediction of probability of a positive ELISA test result (≥ 50 EU in any one of 4 ELISA tests conducted in parallel) for primiparous compared to multiparous cows over 4 years of whole herd testing for MAP infection on a NZ pastoral dairy farm over 4 seasons (2013–2017)

Predicted probability (95% CI) of testing ELISA positive			
Season	Primiparous	Multiparous	*p*-value of difference
2013–2014	15.7% (11.1–22.1)	42.8% (37.1–49.4)	< 0.001
2014–2015	3.8% (2.1–6.8)	15.6% (12.8–19.0)	< 0.001
2015–2016	7.8% (5.0–11.9)	8.1% (6.4–10.3)	0.870
2016–2017	2.6% (1.2–5.3)	2.6% (1.7–4.0)	0.946

### fPCR status

The relationship between the fPCR and ELISA status for the 683 cows tested with fPCR is detailed in Table 6. These 683 cows represent a non-random subset of the population and so are not suitable for further analysis of proportions of the herd. From the 480 cows that were ELISA positive in the 2013–2014, 2014–2015 and 2015–2016 seasons, a total of 455 fPCR results were available. For each year, the denominator was cows testing positive for ELISA and there was no difference (*p* = 0.479) in the proportion of fPCR categories for these ELISA positive cows over the period of study (Table 7). For the cows that were ELISA positive, there was no difference in the unadjusted proportion of primiparous and multiparous fPCR positive cows during the study (Table 8) The results for the GEE model for fPCR status of ELISA positive cows are presented in Table 9.

**Table 6:** fPCR results from cows testing seropositive for JD over 4 years (2013–2017) of whole herd testing in a NZ pastoral dairy farm for MAP infection using 4 ELISA tests conducted in parallel

		fPCR status		
		Not Detected	Moderate	High
ELISA Status	Not Detected	221	5	2
	Low	204	5	2
	Moderate	84	15	3
	High	72	27	8

**Table 7:** Number (proportion; 95% CI) of ELISA positive cows in each fPCR category from 3 years (2013–2016) of whole herd testing on a NZ pastoral dairy farm for *Mycobacterium avium subsp. paratuberculosis* infection using 4 ELISA tests conducted in parallel

fPCR status	Lactation year		
	2013–2014	2014–2015	2015–2016
Not Detected	240 (80.1%; 76.3–85.3)	82 (75.9%; 67.8–84.0)	38 (76.0%; 64.2–87.8)
Moderate	29 (9.8%; 6.4–13.1)	9 (8.3%; 3.1–13.5)	7 (14.0%; 4.4–23.6)
High	28 (9.4%; 6.1–12.8)	17 (15.7%; 8.9–22.6)	5 (10%; 1.7–18.3)

**Table 8:** Number (proportion; 95% CI) of ELISA positive primiparous and multiparous cows subsequently testing fPCR positive for *Mycobacterium avium subsp. paratuberculosis* from a study on a NZ, pastoral dairy farm over 4 seasons (2013–2016)

Number (Proportion; 95% CI) of ELISA positive cows testing fPCR positive			
Season	Primiparous	Multiparous	*p*-value of difference
2013–2014	13 / 39 (33%; 18.5–48.1)	44 / 258 (17%; 12.5–21.6)	0.413
2014–2015	2 / 13 (15%; 0.0–35.0)	24 / 95 (25%; 16.5–34.0)	0.700
2015–2016	5 / 16 (31%; 8.5–54.0)	7 / 34 (21%; 7.0–34.2)	0.500

**Table 9:** Results for the GEE predicting positive fPCR status (> 1 × 10^3^ genomes / mL) for cows that tested positive (≥ 50 EU in any one of 4 ELISA tests conducted in parallel) for antibody to MAP in a study on a NZ pastoral dairy farm over 4 seasons (2013–2017)

Input variable	Coefficient	OR	95% CI	*p*-value^a^
Parity^b^				
Primparous	Ref	Ref		
Multiparous	−0.73	0.48	0.24–0.95	0.026
Study year				
2013–2014	Ref	Ref		
2014–2015	−0.97	0.38	0.07–2.17	0.278
2015–2016	− 0.12	0.89	0.23–3.47	0.869
Interaction^d^				
Multiparous 2014–2015	1.54	4.65	0.76–28.46	0.097
Multiparous 2015–2016	0.46	1.59	0.33–7.82	0.569
ICC^e^		0.110	−0.16 - 0.31	
Variance within		0.113		
Variance among		0.014		

^a^Significance of coefficient

^b^Parity of cow defined as primiparous (≤ 2 years) and multiparous (> 2 years)

^c^Milking season. Each season uses the preceding season as the referent

^d^Interaction term between parity and milking season. Overall significance of the interaction term: < 0.0247

^e^Intra class correlation coefficient

**Fig. 3:**
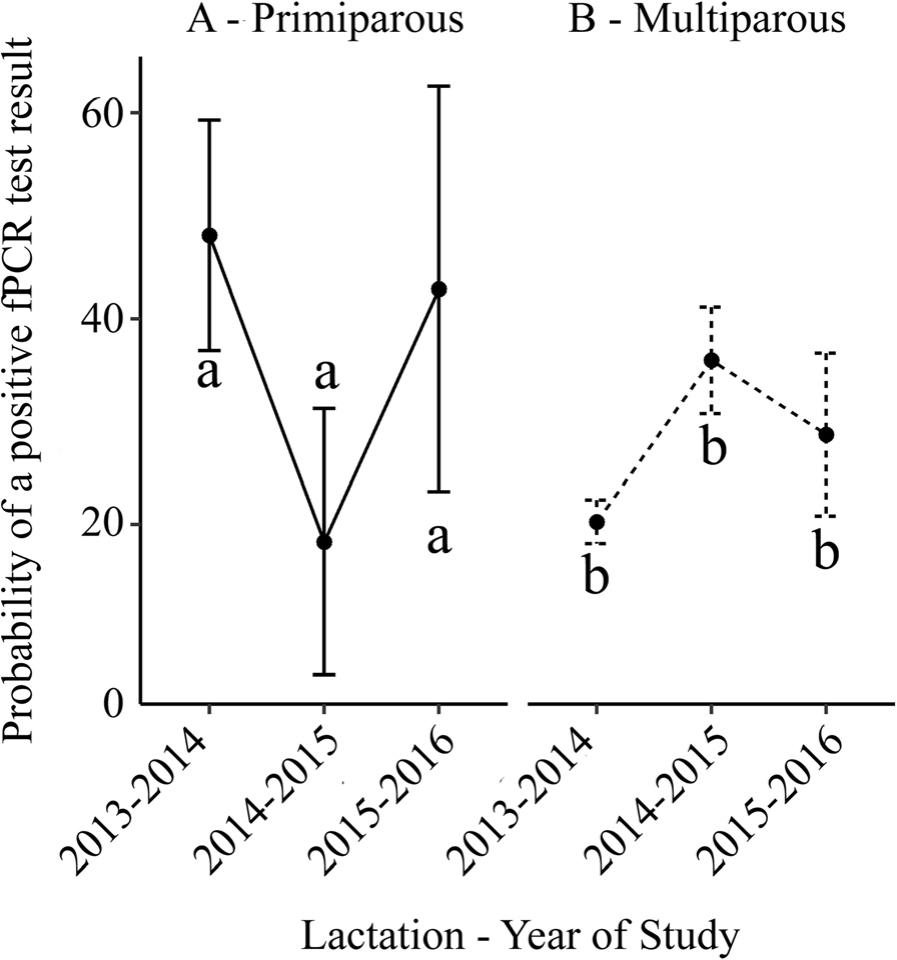
Predicted probability of a positive fPCR test result (> 1 × 10^3^ genomes/mL) for primiparous (left hand panel) and multiparous cows (right hand panel) that tested ≥50 EU in any one of 4 ELISA tests conducted in parallel for antibody to *Mycobacterium avium subsp. paratuberculosis* from a study on a NZ dairy farm over four seasons (2013–2017)

The interaction term predicted differences between primiparous and multiparous cows in the changes in prevalence of fPCR positive status over the 3 years of the study for which data was available. Overall, the interaction term is significant in the model (*p* = 0.0247). The interaction term predicted, for cows testing ELISA positive, the probability of testing fPCR positive was greater for primiparous compared to multiparous cows in 2013–2014 but that there was no difference by age in the probability of testing fPCR positive thereafter. For ELISA positive primiparous cows, there was no difference in the probability of testing fPCR positive by year (*p* > 0.523). A further effect of the interaction was to increase the probability of multiparous cows testing fPCR positive in 2014–2015 compared to 2013–2014 (*p* = 0.08), but there were no other differences by year in the probability for multiparous cows testing fPCR positive. The probability of testing fPCR positive for primiparous compared to multiparous cows that tested ELISA positive is shown in Table 10 and the predicted probability of a positive fPCR for ELISA positive primiparous and multiparous cows in Fig. 3. In Fig. 3, within each parity group, different letters indicate statistically significant differences (*p* < 0.05) between study years. Across the 3 years of the study for which fPCR data was available, there was a significant interaction between parity and year (*p* < 0.024) such that primiparous cows were more likely to be fPCR positive in the 2013–2014 season than in other study years and multiparous cows tended to be more likely to be fPCR positive in 2014–2015 (*p* = 0.08).

**Table 10:** GEE prediction of probability of a positive fPCR test result (> 1 × 10^3^ genomes / mL) for cows that tested positive (≥ 50 EU in any one of 4 ELISA tests conducted in parallel) for antibody to MAP in a study on a NZ pastoral dairy farm over 4 seasons (2013–2016)

Predicted probability (95% CI) of testing fPCR positive			
Season	Primiparous	Multiparous	*p*-value of difference
2013–2014	48.1% (24.2–95.4)	20.3% (14.6–28.1)	0.026
2014–2015	18.3% (3.9–86.1)	35.9% (23.0–56.1)	0.413
2015–2016	42.9% (33.3–88.3)	28.7% (13.0–63.5)	0.580

Of the 297 animals testing seropositive in 2013–2014, 254 were culled over the 4 years of follow up but blood results were available from 43 animals present for the duration of the study. Thirty-one (72%) of these were negative on all subsequent ELISA tests and 12 (28%) were positive on 1 or more tests (9 of these were positive on all subsequent tests). Of the 31 cows that tested ELISA positive in 2013–2014 and negative thereafter, 87% were in the ELISA Low category.

## Discussion

A comparison of the apparent prevalence of MAP infected cows before and after 4 years of the implementation of the control program showed a reduction of MAP infection rate in primiparous and multiparous cows based on a decline in the seroprevalence to MAP antigens detected by ELISA. Year on year, the apparent prevalence of ELISA-positive multiparous animals also fell in this herd and there was a trend for a year on year decline in apparent seroprevalence for primiparous cows. The increase in the apparent seroprevalence in primiparous animals in 2015–2016 compared to the previous year is consistent with this being the last cohort of animals born from untested dams to join the herd.

In this study, not all cows were tested by fPCR so it is not possible to comment on changes in the apparent prevalence of MAP shedding for the herd. For the population of cows that were ELISA positive, there were no changes in the apparent prevalence of fPCR-positive cows in the herd although fecal excretion was more common in primiparous than multiparous cows at the start of the study. The reduction in the conditional probability of positive fPCR status given positive ELISA status of primiparous compared to multiparous cows (Table 10) is also consistent with a reduction in the prevalence of infectious animals in the herd [29].

There was also a year-on year reduction in the culling of suspected clinical cases of JD and a reduction in the proportion of culls testing ELISA or fPCR positive, consistent with a reduction in prevalence of infection in the herd [30].

This was a small-scale case study carried out on a commercial farm without external funding and so resources to complete fPCR on all cows were unavailable; we recognize these necessary limitations of this study and that we have not been able to formally report a reduction in faecal prevalence of MAP. It is noteworthy, however, that in the final year of the study, 116 fecal samples collected from ELISA negative cows representing 10% of the herd, approximately, were also negative for MAP shedding using pooled fPCR. We are not alone in reporting the effects of targeted culling based on antibody prevalence; [31] report the reduction in prevalence of cows testing positive to a milk ELISA in a UK study while other measures of disease prevalence are not reported. Like these authors we have used multiple tests to define our categories. The testing method we used (ELISA tests in parallel followed by fPCR read in series for high ELISA positives) was similar in concept to that recommended for large herds by McKenna et al. [32] and Collins [33]. ELISA testing without confirmatory faecal testing is also used in the Danish JD control programme [34]. Moreover, faecal based methods also have low test sensitivity particularly in the early stages of the disease. As we were preferentially removing animals most likely to be faecal positive the proportion of faecal positive amongst the remaining ELISA low cows would have been very small. In these circumstances a faecal test on the remaining cows would thus have had a poor positive predictive value [35].

In a survey looking at the risk factors for transmission of JD within NZ dairy farms, Soons et al. [36] identified a number of risk factors particular to NZ’s pasture based, seasonal dairy system. These included pooled colostrum feeding, calving cows in large groups, rearing calves in groups, feeding colostrum and milk pooled from many cows to calves and grazing contact between adult cows and calves. One or more of these risk factors were found in over 50% of the herds surveyed (*n* = 427) and all these factors save the last were present on the study farm. The ubiquity of these practices makes many of the herd hygiene measures recommended internationally to control the spread of MAP difficult and unattractive to farmers as managing the risk requires significant investment in additional management systems. Within NZ, there is no coordinated national surveillance or control scheme for JD and dairy industry guidelines designed to help farmers to reduce the impact of JD on farm [37] are similarly compromised by prevailing farming practices.

Animals in their first year are at risk of infection with MAP [38] and this group is considered the most vulnerable age group of animals in MAP control systems. We were unable to protect this cohort of animals as infected dams were not separated from the herd for calving and all calves were fed pooled, antibiotic-contaminated waste milk from cows undergoing treatment. As MAP infected cows are more likely to suffer intercurrent disease such as mastitis [39], this practice will have increased the risk of MAP transmission to calves in this herd.

The decrease in apparent seroprevalence reported in this study occurred despite the farmer being unable to put in place some key biocontainment measures considered necessary to protect new-born and milk-fed calves. This suggests that the culling decisions made removed enough high positive, fecal shedding animals prior to calving to reduce infection of the calf crop year-on-year. Factors that may have mitigated the effect of continued pooled colostrum and milk feeding in the present study may have resulted from the farmer’s willingness to cull large numbers of animals identified in the autumn as JD positive so reducing the pool of JD positive animals at calving in the following spring to much lower levels. This will have decreased the infectious load of MAP to which new born calves were exposed but also have removed a cohort of putatively JD positive calves born to JD positive dams [1].

In the present study, the inability to separate some JD positive dams at calving from the rest of the herd will mean that there may have been leakage of JD positive calves into the replacement calf crop. In a study looking at JD control in American dairy herds, Collins et al. [33] reported an association between the test status of a dam and her offspring in only 1 herd out of the 9 involved in a JD control programme. Miss-identification of dam and off-spring is common in the NZ pastoral system with calves born in large groups of cows, outside and without supervision. Many of the cows in this herd did not have complete herd records on the national database and matching calf to dam was not a management priority at calving. Consequently, no attempt was made to investigate the association between MAP status of calf and dam in this study.

Increasing test sensitivity is an important pre-requisite for effective test and cull control of JD. Lu et al. [40] determined that, in systems where management techniques to control JD were not adopted, testing systems with greater sensitivity and test frequency combined with heavier culling of test positive animals was required to control MAP infection. The role of subsets of infected cattle excreting disproportionately high numbers of MAP, colloquially referred to as supershedders, in the epidemiology of JD has also been highlighted [41]. Working with a single, housed Californian herd of 3,577 cows, Aly et al. (2012) found fecal qPCR testing of ELISA positive cows housed in pens with the highest MAP bioburden to be the most cost-effective method of detecting supershedders. New Zealand’s pastoral dairy systems present no ready equivalent of pen sampling to screen sub-groups for further diagnostic assay, but these workers identified qPCR testing of faecal samples from ELISA positive cows as the next most effective strategy for detecting supershedders.

Necessary and premature culling of cows with clinical JD was a major motivator for the involvement of this farm in the control programme. For the farm, it represented an ongoing loss in value of the culled animals with no apparent beneficial impact on the prevalence of clinical cases. Premature culling associated with JD has been identified as one of the major burdens of the disease [30]. In the current herd, the decision rules in Fig. 1 were used to cull as many reactor animals considered to be at highest risk of MAP infection (High ELISA, fPCR High / Medium) as possible. Selected animals, although clinically normal, were culled at the end of their lactation to maximise their salvage value. Although faecal fPCR positive cows without clinical signs have been shown to weigh approximately 59 kg less at slaughter [42], this represented a considerable saving compared with the clinical losses experienced previously and also ensured that most high-shedding animals did not remain as a source of pseudovertical infection for the next crop of young calves, potentially arresting spread of infection into the future. There is also evidence that MAP infected cows produce less milk even before they develop clinical signs of JD [43] and this effect has been demonstrated previously in this study herd [24].

Although the apparent prevalence of MAP positive cows decreased in this study, differences between MAP strains, environmental challenge and the constraints imposed by the farming system suggest that for other farms a tailored approach is required combining a mixture of testing, culling, management of low-positive cows and improved calf rearing hygiene [44]. Further, it is appreciated that test and cull alone will not eradicate MAP infection or JD and involves ongoing costs for the farmer in terms of regular testing and culling. Moreover, cost-effectiveness of a whole herd test and selective cull strategy measured as the cost per true positive detected will decrease as the programme continues and fewer true positives remain in the herd [6]. In this situation, biennial testing of cows 2–5 years of age that are most likely to be infectious [6] and pooling of samples to reduce cost [25], combined with measures to decrease calf infection, may be appropriate.

Changes in ELISA status of individual cows year on year have also been reported by others [34]. In the current study, 87% of cows that tested ELISA positive in 2013–2014 and subsequently Low were ELISA Low in 2013–2014, supporting the policy of prioritising ELISA Moderate and High cows over ELISA Low cows for culling.

Whilst NZ’s pastoral dairy farming practices may pose some special challenges to JD control, they also have some advantages compared to year-round, confined systems. Seasonal calving means that removing infectious animals at the end of the preceding lactation reduces the infectious pressure at calving which is the key time for the infection of naïve calves [45]. With a combination of diagnostic testing to identify and remove, prior to calving, animals that are the major source of infectious spread, coupled with simple management changes to physically separate replacement calves from MAP infected adult cattle, this study demonstrates that effective reduction in the prevalence of JD is possible for NZ dairy farmers.

## Conclusions

On this farm**,** reduction in the prevalence of infection was achieved by reducing the infectious pressure through targeted culling of heavily shedding animals together with limited measures to protect calves at pasture from exposure to *Mycobacterium avium subsp. paratuberculosis*.

This study demonstrates that - with a combination of pre-calving diagnostic testing to identify and remove animals that are the major source of infectious spread, coupled with simple management changes to physically separate replacement calves from MAP infected adult cattle - effective reduction in the prevalence of JD is possible for NZ dairy farmers.

## Methods

### Study animals

A spring calving, pasture based, Friesian dairy herd (1,250 cows at peak milk) in the South Canterbury region of NZ was selected for the study. In the 5 years preceding this study, the herd had culled annually 3–5% of the milking herd from suspected clinical JD based on clinical signs observed by the owner. In 2009–2010, MAP had been isolated, and JD confirmed histopathologically, from gut and mesenteric lymph node samples from each of 4 cull animals suspected of clinical JD. In 2010–2011, all milking cows over 2 years old were subject to a single serological ELISA (Paralisa™) with fPCR performed on a small subset of the ELISA positive animals. At this test, 97 / 1,086 (8.9%) were ELISA positive and approximately 20% of these ELISA positive cows were shedding high levels (exceeding ≥10,000 genomes / mL) of MAP as determined by fPCR. Considering the high prevalence of ELISA-positive animals the farmer was reluctant to cull all seropositives, most of which appeared healthy and productive. Persistent losses (> 3% pa) of clinically affected animals continued from 2010 to 2013. In 2013–2014 a decision was made to rescreen the herd using serial ELISA, and fPCR testing to identify animals which were shedding high levels of MAP, for culling.

### Johne’s disease control measures

A range of control measures were considered [1] and adapted to accommodate the seasonal breeding and pastoral forage system used routinely in NZ. Uptake of these measures (detailed in Table 2) was recommended to the farmer, but the final decision on which measures were adopted remained with the farmer.

### Sample acquisition and treatment

In the autumn of the 2013–2014, 2014–2015, 2015–2016 and 2016–2017 seasons, a coccygeal tail vein blood sample was collected into a plain blood tube from all milking cows in the enrolled herd. Samples were transported to Disease Research Ltd. (DRL, Mosgiel, NZ) and assayed for circulating antibody to MAP by serum ELISA using a combination of two ELISA tests, Paralisa™ (DRL, Mosgiel, NZ) and IDEXX Paratuberculosis Screening Ab Test (IDEXX Laboratories, Inc., Westbrook, ME, USA). The Paralisa™ methodology was based on previously published procedures for ELISA immunoassays used to diagnose immune reactions to MAP infection in farmed red deer [22]. Besides the IgG_1_ antibody responses to a denatured antigen in the form of Purified Protein Derivative J (PPDj) and a native protein in the form of Protoplasmic Antigen (PPA), an additional MAP-specific recombinant protein antigen, Ag_1_Del_1_, was incorporated into the Paralisa™ test protocol. Final test results were arrived at by considering the antibody level to the IDEXX test and the 3 Paralisa™ test antigens in parallel. IDEXX ELISA assays were performed according to the instructions supplied by the kit manufacturer. Results were classified as follows; for the Paralisa™ serological assays, a classification of Not Detected was returned for results of < 50 ELISA Units (EU) for Johnin, PPA, and Ag_1_Del_1_ antigens, readings of 50–100 EU in any one test were classified as Low, readings of 101–150 EU as Moderate, and readings of > 150 EU as High. For the IDEXX tests, results were classified as Not Detected, Low, Moderate or High based on the response relative to a high positive control. The interpretation of 4 ELISA test results in parallel in the current study increases the sensitivity of the composite ELISA tests to 92%, with a specificity of 59% for detection of ≥1,000 MAP genomes / mL based on a dataset comprising 1,069 matched bovine fecal and peripheral blood samples submitted for routine JD diagnosis [23].

In the autumn of the 2014–2015, 2015–2016 and 2016–2017 season, 7 days after blood sampling, a single fecal sample (10 g approx.) was collected from each cow testing Low, Moderate or High to any of the ELISA tests and forwarded to DRL for quantitative measurement of faecal shedding by fPCR [16, 23, 24]. Briefly, faecal samples submitted for laboratory testing were normalised by gravimetric dilution and homogenised to uniformity. Purified nucleic acids were recovered from 1 ml of normalised faecal homogenates following chemical and mechanical lysis and assayed for the multicopy MAP-specific target gene IS*900* using hydrolysis probe based real time PCR chemistry. PCR amplification efficiency of the diagnostic target was typically > 94%. Quantitation of MAP DNA titer in fecal samples was accomplished using a standard curve comprising DNA dilution standards spanning 7 serial log dilutions of MAP genomic DNA prepared from MAP laboratory strain 316f and results extrapolated and reported as ‘MAP genome copies / mL’ equivalents. DNA standards ranged from 16.5 μg / mL to 1.65 × 10^− 5^ μg / mL; 3 uL of DNA standard was utilized in each 20 uL PCR reaction such that, given a MAP genome size of 4.8 Mbp [46], these values equated to a topmost standard of 1 × 10^7^ genomes / 20uL (5 × 10^8^ genomes / mL) down to a lowermost standard of 10 genomes / 20uL reaction (or 500 genomes / mL). These standards spanned the range of MAP shedding observed in clinical samples and were linear in the assay over the 7 logs (typically *R*^*2*^ = 0.999). Using the qPCR method described, this laboratory has participated in and passed proficiency panels of bovine faecal samples of known infection status and faecal culture titer, administered through the US National Veterinary Services Laboratory (NVSL, Ames, Iowa) as part of an ongoing JD proficiency testing panel for diagnostic laboratories [47]. The NVSL JD proficiency panels are distributed annually to diagnostic and research laboratories both in the US and internationally and are used to accredit testing services for JD diagnostic testing in the US. DRL are a USDA Animal and Plant Health Inspection Service accredited testing laboratory for JD (organism-based methods (direct PCR and pooled PCR) and serum/milk ELISA) and, using this direct qPCR approach, have participated and passed NVSL JD proficiency panels annually since 2008.

Fecal sample data were for this study stratified into shedding categories with MAP shedding scores of ≥1 × 10^3^ to < 1 × 10^4^ genomes / mL classified as Moderate and counts exceeding ≥1 × 10^4^ genomes / mL as High [24]. In this study, fecal samples which returned shedding scores of < 1 × 10^3^ genomes / mL feces were conservatively classified as Not Detected. Classification of MAP status by ELISA and fPCR results is summarized in Table 11.

**Table 11:** Classification scheme of MAP status from ELISA and fPCR results in a study looking at changes in apparent prevalence of MAP infection in a NZ pastoral dairy herd over 4 years of intervention

Test				MAP status
ELISA				
Paralisa™			IDEXX^a^	
Johnin^a^	PPA^a^	Ag_1_Del_1_^a^		
< 50 EU	< 50 EU	< 50 EU	Not Detected	Not Detected
50–100 EU in any test			Low	Low
101–150 EU in any test			Moderate	Moderate
> 150 EU in any test			High	High
fPCR				
< 1 × 10^3^ genomes / mL				Not Detected
≥ 1 × 10^3^ - < 1 × 10^4^ genomes / mL				Moderate
≥ 1 × 10^4^ genomes / mL				High

^a^ELISA results were interpreted in parallel

^b^fPCR results were interpreted in series with ELISA results

### Statistical analysis

The main outcome variable was the apparent JD serological status in the entire adult herd (all animals that had calved at least once) and in first-parity cows over the course of the study [1]. Only those heifers born after full implementation of the control program were used as a cohort for comparison to those born and raised on the farm before implementation of the program and there were no purchased replacements brought into the herd over the study period.

Although JD fecal status was also defined by the fPCR as categorized above, not all animals were tested with fPCR. In each year, only a proportion of cows tested by ELISA were tested with fPCR. All cows that were ELISA positive were tested with fPCR but only a random sample of non-positive ELISA cows were tested with fPCR. These samples were tested as part of a separate study looking at test performance and to be reported. Thus, calculations of sensitivity, specificity and prevalence are not appropriate from this dataset. Furthermore, the cows whose fPCR status was assessed were not a representative sample of the herd. Statistical analysis of the predictors for fPCR status relates only to this non-representative sub population of cows tested with fPCR and was not attempted.

For ELISA status, the predictor variables were days in milk (continuous and rescaled by subtracting the minimum lactation length), parity at sampling date in years, breed (categorical and expressed as ≤50% proportion Friesian genetics, > 50 ≤ 75% Friesian genetics and > 75% Friesian genetics) and study season (categorical). In the secondary analysis of interactions, age was categorized into a dichotomous variable (primiparous and multiparous). In the generalized estimating equation model (GEE), lactation year (2013–2014, 2014–2015, 2015–2016, 2016–2017) was coded using backwards difference coding [48] so that each year was compared to the preceding year.

Database summaries and plots were used to explore the data. All variables were assessed for correlation using a correlation matrix and where a correlation > 0.2 was found, a variance inflation factor to assess collinearity was calculated using auxiliary regressions of one of the correlated variables on the remaining explanatory variables in the model. When the variance inflation factor was > 10, or if when rerunning the model without the variable the remaining coefficients reversed their effect, the collinear variables were assessed for biological plausibility. In this situation, the least useful variable was discarded from the final model. Proportions were compared using a binomial test for large sample sizes.

To model the changes in serological status over time, cows were classified dichotomously as ELISA positive (Low, Moderate or High) or ELISA negative (Table 11). A similar approach was adopted for analysis of the fPCR status of ELISA positive cows with fPCR being dichotomised as cows Not Detected (≤ 1 × 10^3^ genomes / mL) and cows positive (> 1 × 10^3^ genomes / mL). Individual predictor variables were included one at a time in a simple logistic regression model to identify potentially significant predictors (*p* < 0.1). These were then carried forward to a GEE with a binomial distribution and with one data row for each lactation (*n* = 4) for each cow to account for repeat measures. Average marginal probability of a positive ELISA status was calculated with all categorical predictor variables set to zero. In these models, lactation was included as a repeated effect within cow identity. The proportion of the total variance for repeat measures on the same cow was calculated as the intra-class correlation coefficient (ICC). To account for repeated measures within an individual, various covariance structures (autoregressive, exchangeable and independent) were added. To assess the appropriateness of the chosen correlation structure, the quasi-likelihood under the independence model criterion statistic [49, 50] was calculated. Given that the number of clusters was small an F distribution was used to calculate the *p*-values for group variables and a t-distribution for single variables [51]. In the model, to assess whether a variable was acting as a confounder on the outcome, the crude estimate of each variable was compared with the adjusted estimate after inclusion of the potential confounder. If the ratio between the difference of the crude estimate and the adjusted estimate of the effect of the variable differed by > 10% the additional variable was designated as a potential confounder.

Once all potential confounders had been identified they were placed into the model along with all two-way interactions between status and the confounder. Each non-significant interaction term (*p* > 0.05) was removed one at a time and the model re-run until no non-significant interaction terms remained. At this point, all two-way interactions were assessed between the other remaining variables and excluded if *p* > 0.05. Given that there were only a small number of variables a hand-built model was constructed. All analysis was conducted using R [52].

## Data Availability

An anonymized form of the datasets used and/or analyzed during the current study are available from the corresponding author on reasonable request.

## Abbreviations

DRL: Disease Research Ltd
EU: ELISA Units
fPCR: fecal PCR
GEE: Generalized Estimating Equation
ICC: Intra-Class Correlation Coefficient
JD: Johne’s disease
MAP: *Mycobacterium avium subsp. paratuberculosis*
NZ: New Zealand
OR: (Odds Ratio)
PPA: Protoplasmic Antigen
PPDj: Purified Protein Derivative J
RR: Relative Risk
